# Coexisting Organoaxial Gastric Volvulus and Sigmoid Volvulus With Complications

**DOI:** 10.14309/crj.0000000000001458

**Published:** 2024-08-22

**Authors:** Mariyam Sadikot, Gunter Nicolle

**Affiliations:** 1 Department of Family Medicine, Lee's Summit Medical Center, Lee's Summit, MO

**Keywords:** gastric volvulus, sigmoid volvulus

## Abstract

Very few cases of coexisting organoaxial gastric and sigmoid volvuli have been reported. In here we describe 1 such case. Our patient, a 92-year-old man, was treated with a flexible sigmoidoscopy with decompression for his sigmoid volvulus. For the gastric volvulus, the patient was initially treated with an upper endoscopic gastric decompression and subsequently treated with a laparoscopic anterior gastropexy. After the gastropexy, the patient developed bleeding and ischemia of the stomach, which is a rare complication due to the stomach's rich blood supply. This case report reiterates the need to consider rare complications in patients with such rare pathologies.

## INTRODUCTION

The presence of volvuli affecting different parts of the gastrointestinal tract simultaneously is a rare occurrence.^1^ Volvuli is twisting or torsion of a segment of the alimentary tract. Organo axial gastric volvulus occurs when stomach rotates around its longitudinal axis. Similarly, organoaxial sigmoid volvulus occurs when the colon rotates around its longitudinal axis with only 1 transition point and no closed loop obstruction.^2^ To our knowledge, there are only 4 known cases of coexisting organoaxial volvulus of the stomach and sigmoid colon.^1,3^ Out of the 4, the complete case reports for 2 of the cases could be located. Here, we describe the fifth reported case of a 92-year-old man with coexisting gastric and sigmoid volvuli.

## CASE REPORT

The patient, a 92-year-old man, presented at the emergency department with acute-onset epigastric pain and distention of the abdomen. He had a medical history of paroxysmal atrial fibrillation and was on the anticoagulant apixaban. The anticoagulant was changed to enoxaparin, a short-acting anticoagulant, on admission. The patient had also been treated for colon cancer with a surgical resection 13 years ago, coronary artery disease with a coronary artery bypass graft 8 years ago, and had been treated for B-cell lymphoma with chemotherapy 2 years prior.

**Figure 1.**
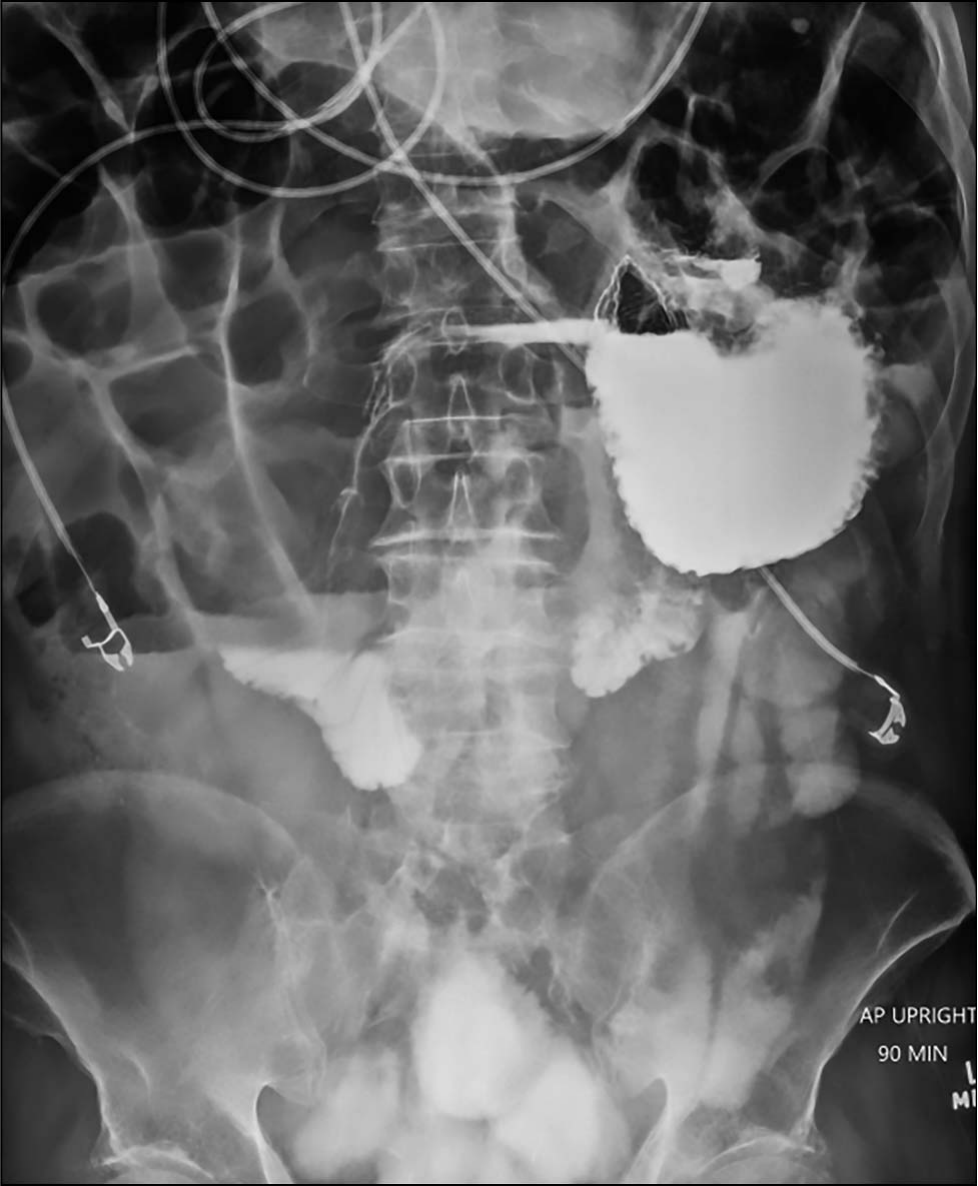
Stomach appearing flipped with respect to the duodenum suggestive of a gastric volvulus.

**Figure 2.**
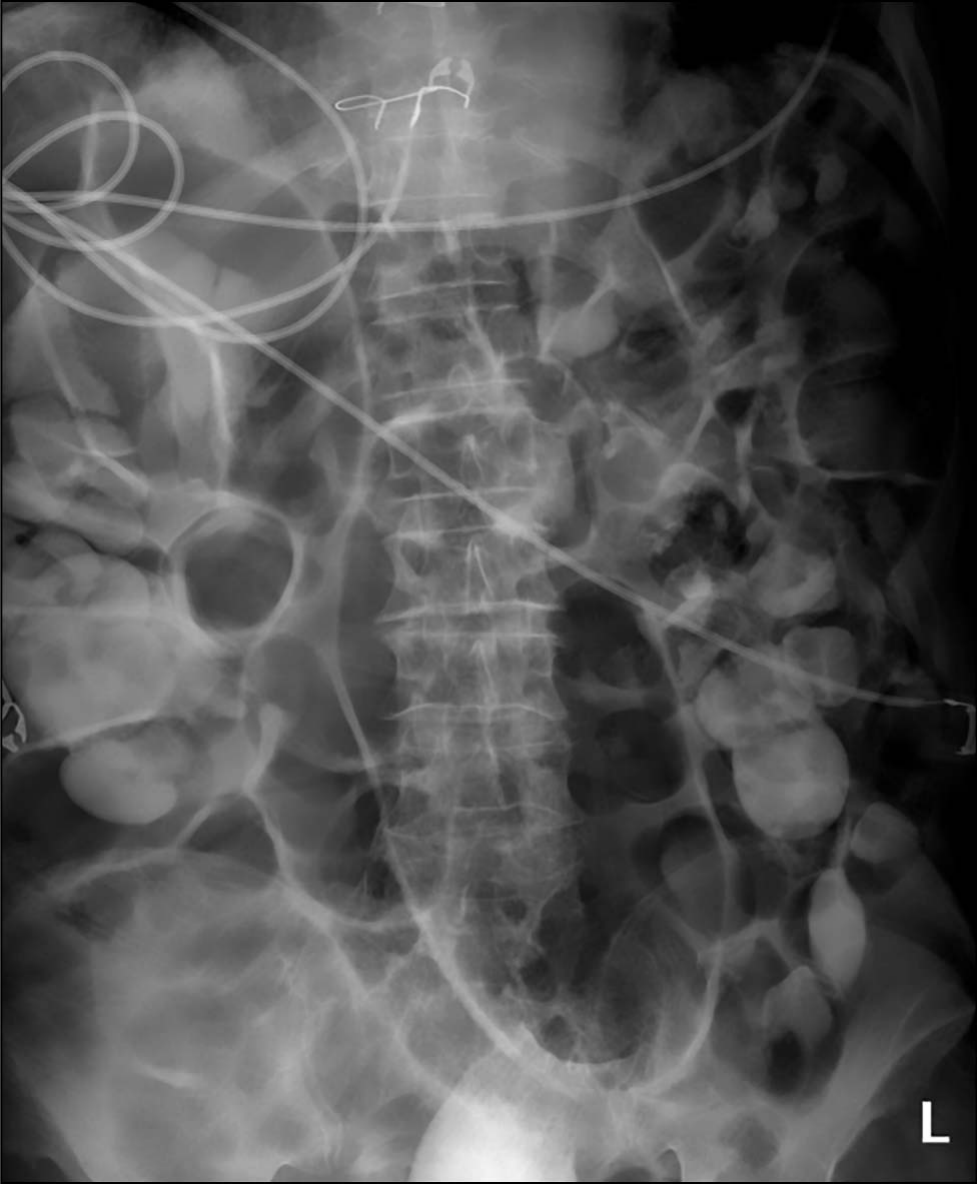
Plain radiograph showing a “coffee bean” sign suggestive of organoaxial sigmoid volvulus.

On arrival at the emergency department, the patient presented with acute-onset epigastric pain and distention of the abdomen. A computed tomography scan with contrast of the abdomen and pelvis showed a small bowel obstruction with a transition point at the small bowel colonic anastomosis and a possible internal hernia. The patient drained 500 mL of bilious gastric fluid initially through a nasogastric tube. A small bowel gastrografin study was performed, in which the stomach appeared flipped with respect to the duodenum, suggestive of a gastric volvulus. An abdominal x-ray showed a distended sigmoid colon with twisting in the rectosigmoid. The ascending and descending colon was entirely distended with contrast and air. These findings were consistent with an obstructing sigmoid volvulus (Figures 1 and 2).

An endoscopy-assisted decompression and flexible sigmoidoscopy was performed on day 3 of admission. A gastric volvulus without any evidence of ischemia or necrosis along the stomach lining was identified and decompressed during the endoscopy. The flexible sigmoidoscopy identified a sigmoid volvulus which was then successfully decompressed. Afterward, a liquid diet was started; however, abdominal distension reoccurred. An abdominal x-ray showed distended small and large loops of the bowel. A da Vinci-assisted diagnostic laparoscopy with colonic decompression on day 5 of admission was performed. During the surgery, a dilated stomach with no evidence of gastric volvulus was seen. However, as the upper endoscopy had identified a gastric volvulus, a decision was made to proceed with a gastropexy and the stomach was sutured to the anterior abdominal wall. The colon did not have any twisting but was dilated. Owing to this, a rectal tube was placed up to the descending colon with low intermittent suction applied for decompression.

On postoperative day (POD) 1, the patient passed flatus and tolerated a liquid diet with no apparent complications. On POD 2, the patient developed distention, nausea and vomiting. The patient also showed signs of tachycardia, fever, and leukocytosis. Based on these findings, antibiotics and fluids were initiated. An abdominal x-ray showed significant gaseous distention, which may have been a result of colonic pseudo-obstruction or intermittent sigmoid volvulus. A nasogastric (NG) tube was placed, after which the patient had bilious gastric output. From POD 3 to 5, the patient was started on a liquid diet but continued to have output through the NG tube and concurrent obstipation.

On POD 6, the patient had an episode of hematemesis and subsequently a constant intravenous pantoprazole and fluid drip was initiated. Owing to this episode, the anticoagulants were stopped on POD 6. An upper endoscopy identified clotted blood in the esophagus, a deformity of the gastric body suggestive of ischemia and a Mallory-Weiss tear with stigmata of recent bleeding. The active bleeding was stopped with a hemostatic spray. The patient remained intubated after the procedure. Within a few hours, the patient became hemodynamically unstable with tachycardia and hypotension. The presence of blood in the NG tube was observed, suggestive of active bleeding. Packed red blood cells, fluids, and fresh frozen plasma were administered. A plan was proposed to get the computed tomography angiography of the chest, abdomen, and pelvis to locate the bleeding source, followed by possible embolization. However, the family of the patient decided to proceed with comfort care given the patients advanced age.

## DISCUSSION

Gastric volvulus is a rare condition that was first described by Berti on postmortem examination in 1866.^4^ A gastric volvulus may be a primary volvulus, caused due to the laxity of gastric ligaments, or may be secondary to anatomical defects like diaphragmatic or hiatal hernias.^5^ In terms of orientation, a gastric volvulus can be organoaxial in which the stomach rotates around the longitudinal axis or mesenteroaxial in which the stomach rotates along the greater or lesser curvatures.^6^ Acute gastric volvulus is considered a surgical emergency due to its high mortality rate. Open surgical reduction used to be the most common method for reduction of gastric volvuli, but endoscopic and laparoscopic approaches are now preferred due to recent advances.

Sigmoid volvulus is the wrapping of the sigmoid colon around its axis, which leads to obstruction. Multiple hypotheses for the cause of sigmoid volvulus exist. One common hypothesis is that a diet rich in fiber favors formation of a dolichosigmoid, which is the elongation of the sigmoid colon with regard to its narrow mesenteric attachment.^7^ Another theory is that sigmoid volvuli occur due to chronic constipation and associated elongation and dilation of the colon. This can explain its increased occurrence in old, institutionalized adults with chronic constipation. The mortality rate in sigmoid volvulus depends on whether the volvulus causes obstruction, the timeline of interventions, and the functional status of the patient but is generally less than that of gastric volvuli. The treatment of sigmoid volvulus generally consists of endoscopic decompression.^8^

In our case, similar to the previously reported cases, the 2 volvuli were treated independently of each other despite simultaneous existence. In all cases including ours, anterior gastropexies were performed to successfully treat the gastric volvulus. In addition, this case was the only 1 to concurrently perform a endoscopic decompression for the gastric volvulus.

To treat the sigmoid volvulus, a sigmoidoscopy and decompression with a flatus tube was performed, similar to 1 of the previous cases. Another case reported performing a sigmoid colectomy with colocolic anastomosis due to the volvulus' recurrence. In 1 of the cases, it was suggested that angulation and distortion of the stomach may have been responsible for development of a gastric ulcer. In contrast, in our case, the patient developed ischemia and bleeding of the stomach after gastropexy, which itself is a rare complication.^9,10^ While the source of the bleeding could not be definitively ascertained, possible sources of bleeding may be the development of a gastric ulcer, as reported in the previous case, or even an ulcer at the gastropexy site (which can lead to the involvement of the superior gastric artery) as reported in another case of anterior gastropexy.^11^

The cause for increased susceptibility to multiple volvuli remains unidentified. In this case, the patient exhibited a significant amount of air in both the small and large intestines, resulting in their dilation, a condition known as aerocoly. Two of the previously identified cases of multiple volvuli have suggested that aerocoly may be a cause of the multiple volvuli; however, no definitive pathophysiological evidence has been identified.^1^ This case offers another data point linking aerocoly to multiple volvuli.

There were no other common features identified between the other reported cases of multiple volvuli which could be indicative of increased susceptibility to multiple volvuli. Ischemia of the stomach, a rare complication itself, was also only reported in this case of multiple volvuli. Thus, multiple volvuli and other rare complications must be kept in mind in cases of suspected gastric or sigmoid volvuli. Generalizing this finding, this case highlights the need to keep in mind rare complications which may occur after treating rare conditions.

## DISCLOSURES

Author contributions: M. Sadikot: manuscript revision, literature review, and manuscript writing. G. Nicolle: conception, critical analysis, revision and final approval, and is the article guarantor.

Financial disclosure: This research was supported (in whole or in part) by HCA Healthcare and/or an HCA Healthcare affiliated entity.

Informed patient consent was obtained for this case report.

The views expressed in this publication represent those of the author(s) and do not necessarily represent the official views of HCA Healthcare or any of its affiliated entities.
